# Supplementary material for: Exploring the potential biomarkers for prognosis of glioblastoma via weighted gene co-expression network analysis
Source: PeerJ. 2022 Jan 18;10:e12768. doi: 10.7717/peerj.12768 (PMC8781321; doi:10.7717/peerj.12768)
Supplement: Supplemental Information 3 [file peerj-10-12768-s003.zip › ID TCGA.docx]

ID

TCGA-27-2519 TCGA-06-5858 TCGA-26-5136 TCGA-06-0141 TCGA-27-1831 TCGA-12-3653 TCGA-06-0125 TCGA-06-0882 TCGA-06-0747 TCGA-06-0211 TCGA-12-3652 TCGA-15-1444 TCGA-32-4213 TCGA-02-0055 TCGA-06-0157 TCGA-06-0750 TCGA-12-0821 TCGA-41-5651 TCGA-27-1830 TCGA-06-0749 TCGA-76-4928 TCGA-06-0210 TCGA-06-0745 TCGA-32-2615 TCGA-28-5215 TCGA-28-5220 TCGA-32-1970 TCGA-06-5410 TCGA-27-2521 TCGA-14-0787 TCGA-06-0878 TCGA-41-2572 TCGA-26-5134 TCGA-12-0618 TCGA-02-2485 TCGA-26-5133 TCGA-14-0789 TCGA-06-0139 TCGA-06-2558 TCGA-26-5132 TCGA-06-0129 TCGA-06-5411 TCGA-28-5209 TCGA-27-1837 TCGA-12-5299 TCGA-76-4932 TCGA-06-2564 TCGA-26-1442 TCGA-41-4097 TCGA-28-2514 TCGA-06-0171 TCGA-06-2562 TCGA-28-2513 TCGA-06-1804 TCGA-06-0645 TCGA-32-1980 TCGA-12-0616 TCGA-06-2563 TCGA-06-0219 TCGA-41-3915 TCGA-28-5207 TCGA-28-1747 TCGA-19-4065 TCGA-15-0742 TCGA-19-2624 TCGA-02-0047 TCGA-27-1835 TCGA-14-0781 TCGA-06-0649 TCGA-06-5856 TCGA-14-2554 TCGA-06-0130 TCGA-14-1823 TCGA-06-0187 TCGA-76-4926 TCGA-19-1390 TCGA-12-5295 TCGA-08-0386 TCGA-06-0221 TCGA-06-5417 TCGA-14-0817 TCGA-06-0156 TCGA-27-2528 TCGA-06-0190 TCGA-28-5216 TCGA-32-2616 TCGA-06-2570 TCGA-19-1389 TCGA-14-1402 TCGA-14-0871 TCGA-14-1034 TCGA-32-2638 TCGA-26-5139 TCGA-06-0132 TCGA-06-5418 TCGA-19-2620 TCGA-02-2486 TCGA-16-0846 TCGA-19-2619 TCGA-06-0744 TCGA-06-2561 TCGA-28-1753 TCGA-06-0174 TCGA-02-2483 TCGA-19-1787 TCGA-28-2509 TCGA-26-5135 TCGA-19-5960 TCGA-32-1982 TCGA-76-4931 TCGA-32-5222 TCGA-06-0152 TCGA-28-5208 TCGA-28-5213 TCGA-06-0743 TCGA-27-2524 TCGA-06-5408 TCGA-06-2565 TCGA-06-5413 TCGA-32-2632 TCGA-06-0138 TCGA-06-2559 TCGA-06-5412 TCGA-06-0686 TCGA-12-0619 TCGA-16-1045 TCGA-32-2634 TCGA-06-0644 TCGA-14-0790 TCGA-06-0238 TCGA-06-2557 TCGA-14-1829 TCGA-06-0184 TCGA-76-4925 TCGA-06-0168 TCGA-19-2629 TCGA-06-5414 TCGA-41-2571 TCGA-27-2523 TCGA-76-4929 TCGA-19-2625 TCGA-06-2567 TCGA-12-3650 TCGA-27-1834 TCGA-28-5218 TCGA-27-2526 TCGA-14-1825 TCGA-06-0178 TCGA-06-0158 TCGA-27-1832 TCGA-06-5416 TCGA-19-0957 TCGA-14-0736 TCGA-28-5204 TCGA-06-5859 TCGA-76-4927 TCGA-12-1597 TCGA-06-0646 TCGA-06-2569
